# General Intelligence

**Published:** 1845-01

**Authors:** 


### GENERAL INTELLIGENCE.

*Dedication of the Edifice of the Rush Medical College.*—The ceremonies upon this occasion took place on the evening of Friday, December 18th; the severe weather of early autumn having delayed the completion of the building until that time.

The lecture-room upon the first floor was crowded to excess with the sage and the gay. An appropriate prayer, by the Rev. Mr. Patterson, commenced the services of the evening. The address usual upon such occasions was delivered by D. Brainard, M.D., President of the Faculty, and Professor of Anatomy and Surgery. We give below an abstract, which, however, can give but a limited idea of the beauty and force of the Doctor's remarks:

He spoke first of the history of the school and of its rapid progress, which already permitted its friends to assemble in so fine an edifice. He next proceeded to consider the reasons why institutions of the higher order should be planted in the West; said that it was soon to be the seat of power for the whole northern continent. He then alluded to some popular errors concerning education, and showed that it was more important and difficult to produce a few men who make discoveries and advance the knowledge of mankind, than a much greater number who are incapable of it. He advanced the position that this could only be done by time and the coöperation of many persons, and traced the history of some of the celebrated colleges of Europe, to show the successive degrees by which they have become perfected.

He then proceeded to show the manner in which they should be conducted in this country,—that they should be regarded as instituted exclusively for the public good, and not for the benefit of individuals, and assured his hearers "that this edifice, which had been erected by their liberality, should be forever sacred to the cause of science and humanity." He then traced the plans for its future improvement; for the formation of a library; of a museum, and the establishment of a hospital, which should place it on a level with the first institutions in the country.

He next addressed himself to the pupils, congratulated them upon the prospects opening before them in the West, urged them to prepare themselves for the first places in the profession, assuring them that their destinies in life would be greatly influenced by their present plans and aspirations.

The discourse concluded with congratulations to the friends of the institution on its success and prospects, and thanks for their liberality; and with the assurance that whatever obstacles threatened to obstruct its progress they would be overcome; that its Faculty would be devoted to it, to the degree necessary to ensure its prosperity. Ed.

